# Molecular diagnosis of genital tract infections among HIV-positive women in Iran

**Published:** 2018-08

**Authors:** Mohammad Amin Behzadi, Mohammad Ali Davarpanah, Mandana Namayandeh, Bahman Pourabbas, Soheyla Allahyari, Mazyar Ziyaeyan

**Affiliations:** 1 Clinical Microbiology Research Center, Shiraz University of Medical Sciences, Shiraz, Iran; 2 HIV Research Center, Shiraz University of Medical Sciences, Shiraz, Iran

**Keywords:** Human papilloma virus, Herpes simplex virus, *Chlamydia trachomatis*, *Neisseria gonorrhoeae*, HIV

## Abstract

**Background and Objectives:**

Human immunodeficiency virus (HIV)-infected women are usually at a higher risk of sexually transmitted infections (STIs) than others. The objective of this study was to characterize the prevalence of human papilloma virus (HPV), herpes simplex virus (HSV), *Chlamydia trachomatis* (CT), and *Neisseria gonorrhoeae* (NG), and associated risk factors among HIV-infected women in Fars province, Iran.

**Materials and Methods:**

In this cross-sectional study, cervical swab samples were collected from 71 HIV-infected women, aged 17–45 years (mean ± standard deviation: 31.11 ± 6.58 years), and tested for HPV, HSV, CT, and NG using PCR assays.

**Results:**

Overall, 77.5% of patients were positive for the tested STIs with the following distribution: 36 (50.7%) HPV, 7 (9.9%) HSV, 4 (5.6%) NG, and 27 (38%) CT. From those, 39 (55%) were positive for only one infection, while 16 (22.5%) were positive for multiple infections. We observed that the prevalence of all tested STIs increased by age, except for HSV which showed a slight decrease, although not statistically significant. Socio-economic factors such as low educational level, multiple sex partners, and being a sex worker significantly correlated with higher positive prevalence of STIs in the studied population.

**Conclusion:**

A high prevalence of STIs was observed among HIV-infected women in this region. These data might prompt policy makers and STI experts to focus on providing a comprehensive sex education, including participation in screening programs for STIs among high-risk groups.

## INTRODUCTION

Human immunodeficiency virus (HIV)-infected women are usually at a higher risk of sexually transmitted infections (STIs) than others. The human papilloma virus (HPV), herpes simplex virus (HSV), *Neisseria gonorrhoeae* (NG) and *Chlamydia trachomatis* (CT) are the most common agents involved in genitourinary tract infections in HIV-positive patients (1). The clinical signs of infection can widely vary from no overt disease to severe symptoms. In HIV-infected patients with a concomitant HPV infection, the associated cervical abnormalities are more prevalent, and the lesions can become aggressive leading to more complicated treatments (2). Recent reports have shown that HSV-1 and HSV-2 infections can trigger the reactivation of Kaposi’s Sarcoma-associated Herpes virus from latency and may function as a cofactor to develop disease (3, 4). CT and NG are also among the bacterial pathogens that can cause serious sequelae in HIV-positive women, and screenings for these agents are recommended to help reduce the incidence of complicated infections such as pelvic inflammatory disease (5).

There is a direct relationship between STIs and genital HIV shedding in HIV-positive women (6), and such co-infections may facilitate the sexual transmission of the virus and accelerate disease progression (7). Appropriate treatment of NG and CT infections as well as suppressive therapy of HSV and HPV infections can help reduce the virus load in the genital tract of the HIV patients (8).

Longevity and improved quality of life in HIV-positive patients under anti-retroviral therapy has changed the landscape of diseases observed among these patients (9, 10). For example, it is estimated that up to 40% of the HIV-infected individuals may develop a neoplastic lesion, including HPV related cervical cancer (11, 12). Therefore, the prevalence of STI co-infection in HIV patients is a risk factor for development of secondary genital infections that can be life-threatening. The epidemiology of such infections among HIV patients is poorly defined in much of Middle East, including Iran. Hence, the objective of the present study is to investigate the prevalence of HPV, HSV, CT and NG, and associated socio-economic risk factors among HIV-infected women referred to the Lavan outpatient behavioral clinic in Fars province, Iran.

## MATERIALS AND METHODS

### Institutional ethical approval.

The study protocol was approved by the research co-ordinating committee at the Clinical Microbiology Research Center, Shiraz University of Medical Sciences. Written informed consents were obtained from the women enrolled in the study.

### Study population.

This study included all HIV-infected women (N=71, ages ranging between 17 and 45 years (mean ± standard deviation: 31.11 ± 6.58 years)) who attended the Lavan outpatient behavioral clinic affiliated with Shiraz University of Medical Sciences, Iran, during 2015–2016. The patients were referred for a routine medical visit to receive medical advice as well as their HIV medication. Inclusion criteria were HIV infection and symptoms such as itching in the genital area, dyspareunia, dysuria, or abnormal vaginal discharges in the past 6 months. Exclusion criteria were pregnancy or bleeding per vagina. Based on blood CD4+T-cell levels, none of the patients had developed AIDS at time of sampling (CD4+ T-cell count less than 200 mm^3^ was considered as AIDS). The studied population was divided into four age groups; 17–23 (I), 24–30 (II), 31–37 (III), and 38–45 (IV) years old. At time of sampling, none of the women were under treatment for cervical intraepithelial neoplasia (CIN), nor did they have a recent history of CIN. Each patient was interviewed by a female health professional staff to collect relevant socio-economic information including demographics (age), educational status, life-style behavior (drug addictions), and sexual activity (marital status, number of sex partners, using condom, and exchange of sex for money). Swab samples were collected from cervix and the cervical os by a trained physician. The swabs were placed in transport medium tubes, kept at 4°C for a maximum of 6 h and then transferred to the Clinical Microbiology Research Centre for laboratory analysis. At the laboratory, the tubes containing the samples were vigorously vortexed, the swabs were discarded, and the samples were stored at −70°C until examination.

### DNA extraction.

DNA was extracted from 200μl of viral transport medium of all swab samples using the QIAamp DNA Blood Kit (Qiagen, Hilden, Germany), according to the manufacturer’s Blood and Body Fluid Spin Protocol. During the extraction process, negative and positive controls were included between every 20 clinical samples. For detection of HPV, HeLa and Vero cell lines, spiked into each collection medium, were used as positive and negative extraction controls, respectively, in each extraction batch (13). The clinical isolates of HSV-1 and HSV-2, which had been verified and typed by direct immune-fluorescent assay, were used as positive controls for HSV. To detect NG and CT, a standardized amount of internal control DNA, supplied with the real-time PCR kit, was added to the lysis buffer kit to monitor the efficiency of extractions.

### Human papilloma virus PCR.

To detect HPV in the cervical samples, a nested PCR was adapted by using two consensus primers (MY09/MY11, GP5+/GP6+) to amplify a broad-spectrum of HPV geno-types (14). Briefly, the first round of PCRs for generic HPVs was performed in a Veriti 96-well thermal cycler instrument (Applied Biosystems, Foster City, CA, USA) using 2× AmpliTaq Gold Fast PCR Master Mix (Applied Biosystems, Foster City, CA, USA) and MY11/MY09 primers. The first round PCR product was subsequently subjected to a second round of PCR using 2× Power SYBR Green PCR Master Mix (Applied Biosystems, Warrington, UK) and GP5+/GP6+ primers. The real-time PCR was performed using the One-step plus Sequence Detection System (Applied Biosystems, Foster City, CA, USA), following which a dissociation curve was constructed in the range of 55°C to 95°C and the data were interpreted using melting curve analysis.

### Herpes simplex viruses TaqMan real-time PCR.

HSV DNA was detected by the TaqMan real-time PCR method using a previously described primer set and probe (15). The primers amplify a 92 bp fragment within a highly conserved region of the DNA polymerase gene from the HSV-1 and HSV-2. Amplification was performed using TaqMan Gene Expression Master Mix Reagents (Applied Biosystems, Foster City, CA, USA) in an Applied Biosystems Sequence Detector 7500 machine (Applied Biosystems, Foster City, CA, USA).

### *Neisseria gonorrhoea* and *Chlamydia trachomatis* TaqMan real-time PCR.

Commercially available TaqMan real-time PCR kits were used to detect NG and CT in the clinical specimens. All the procedures were performed according to the directions and recommendations in the manufacturer’s protocols (*Neisseria gonorrhoea* and *Chlamydia trachomatis* standard kits; Primer Design Ltd., Millbrook Technology Campus, Southampton, UK). The amplification was performed using the TaqMan Gene Expression Master Mix Reagents (Applied Biosystems, Foster City, CA, USA) in a 7500 Real-Time PCR System instrument (Applied Biosystems, USA).

### Statistical analysis.

The statistical analyses were done by SPSS for Windows (version 16, SPSS Inc., Chicago, IL, USA) and the data were considered statistically significant at a two-sided p< 0.05. Differences in prevalence data of the four studied STIs (HPV, HSV, CT and NG) between age groups were analysed using analysis of variance (ANOVA) and chi-square tests. Moreover, demographic and behavioural characteristic of participants were also calculated and compared between STIs and non-STI groups. All comparison graphs were generated using GraphPad Prism version 5.0 (GraphPad Software Inc., CA, USA).

## RESULTS

**Fig. 1.**
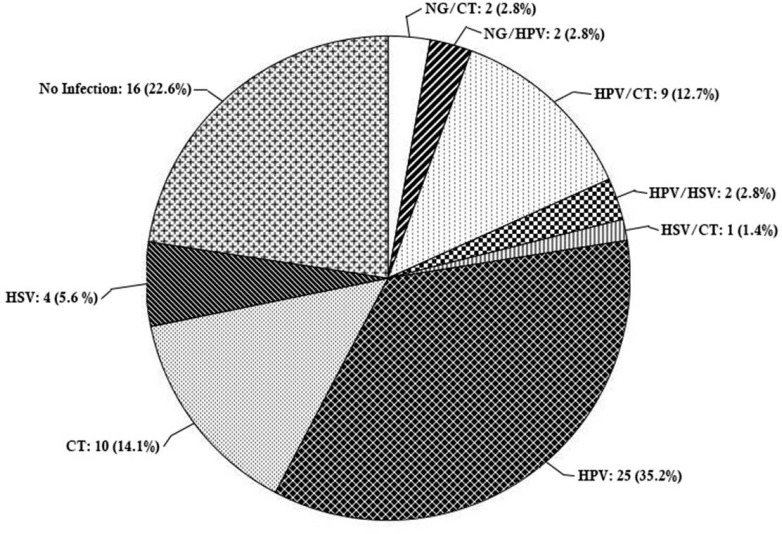
Distribution pattern of human papilloma virus (HPV), herpes simplex virus (HSV), *Neisseria gonorrhoeae* (NG) and *Chlamydia tracomatis* (CT) infections and respective co-infection among HIV-infected women in Shiraz, Iran (n=71).

**Fig. 2.**
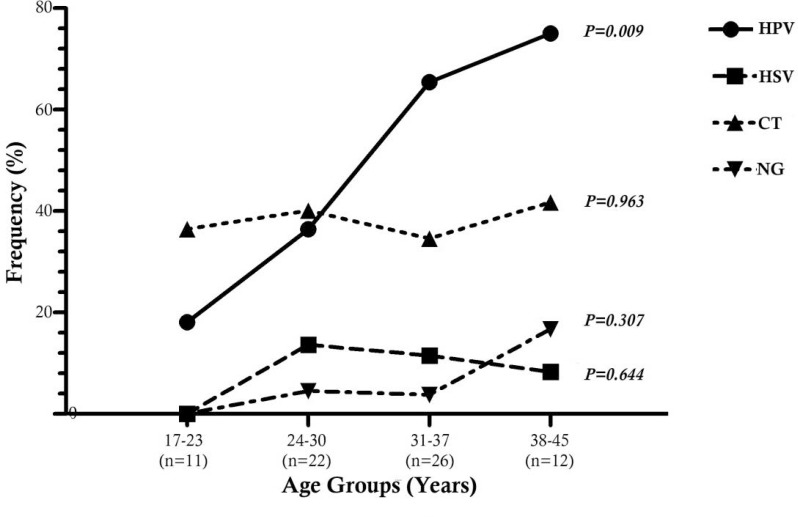
Trend in prevalence of human papilloma virus (HPV), herpes simplex virus (HSV), Neisseria gonorrhoeae (NG) and Chlamydia tracomatis (CT) infections among HIV-infected women by age; Shiraz, Iran (n=71).

**Fig. 3.**
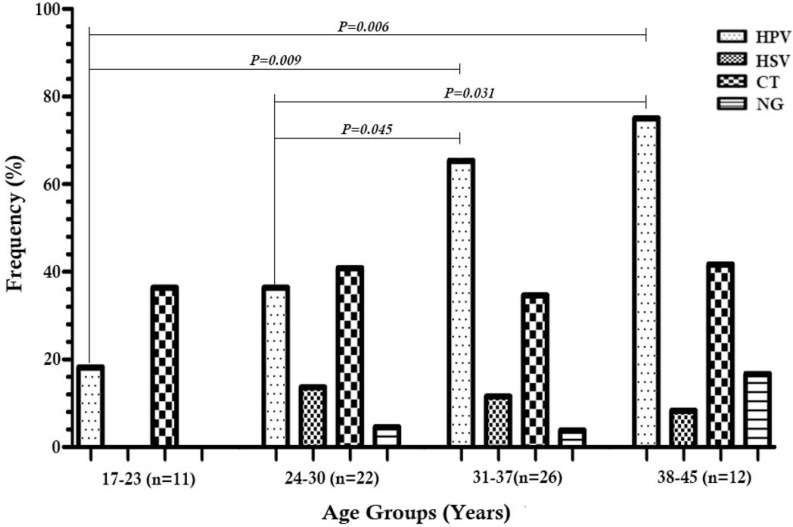
Prevalence of human papilloma virus (HPV), herpes simplex virus (HSV), *Neisseria gonorrhoeae* (NG) and *Chlamydia trachomatis* (CT) infections in different age groups among HIV-infected women in Shiraz, Iran (n=71).

Of the 71 clinical samples collected, the following distributions were observed among patients with positive PCR results: 36 (50.7%) HPV, 7 (9.9%) HSV, 4 (5.6%) NG, and 27 (38%) CT. The prevalence of STIs was 77.5 %, in which 39 (55%) of the patients had only one infection, whereas 16 (22.5%) patients had multiple infections (Fig. 1). The prevalence of any of the four STIs increased by age, except for HSV which showed a slight decrease in the groups III and IV (Fig. 2), although not statistically significant. There were significant differences in prevalence rates of HPV between age groups I and III (p=0.009), I and IV (p= 0.006), II and III (p=0.045), and II and IV (p=0.031, Fig. 3).

The descriptive data and the comparison of respective risk factors between the patients with and without STIs are presented in Table 1. The statistical analyses indicate significant differences in educational status, number of sex partners, and number of patients who exchange sex for money between the STI and non-STI groups.

**Table 1.** Comparison of behavioral and demographic characteristics between HIV-infected women with or without STIs in Shiraz, Iran (n=71).

**Characteristic**	**Total count (%) n=71**	**STIs Patients (%) n=58**	**Non-STIs Patients (%) n=13**	***P*-value**
Education				
Illiteracy	2 (2.8)	2 (3.4)	0 (0)	0.035
Primary	23 (32.4)	22 (37.9)	1 (7.7)	
Middle school	32 (45.0)	21 (36.2)	11 (84.6)	
High school	10 (14.1)	9 (15.5)	1 (7.7)	
University	4 (5.7)	4 (6.9)	0 (0)	
Marital status				
Never married	0 (0.0)	0 (0.0)	0 (0.0)	0.19
Married	50 (70.4)	38 (65.5)	12 (92.3)	
Divorced	4 (5.7)	4 (6.9)	0 (0.0)	
Temporary marriage	5 (7.0)	4 (6.9)	1 (7.7)	
Widowed	12 (16.9)	12 (20.7)	0 (0)	
No. of sex partners				
1	38 (53.5)	27 (46.5)	11 (84.6)	0.038
2–5	23 (32.4)	21 (36.2)	2 (15.4)	
>5	10 (14.1)	10 (17.3)	0 (0.0)	
Using condom				
Never	9 (12.7)	8 (13.8)	1 (7.7)	0.300
Almost	41 (57.7)	31 (53.4)	10 (76.9)	
Always	21 (29.6)	19 (32.8)	2 (15.4)	
Exchange of sex for money				
Never	50 (70.4)	37 (63.8)	13 (100.0)	0.010
Almost	21 (29.6)	21 (36.2)	0 (0.0)	
Drug addiction				
Yes	13 (18.3)	13 (22.4)	0 (0.0)	0.059
No	58 (81.7)	45 (77.6)	13 (100.0)	

## DISCUSSION

In the present study, four major STIs were investigated in 71 HIV-infected women who were attending an outpatient behavioral clinic in Fars province, Iran. The main findings of this study highlight the high prevalence (77.5%) of STIs in HIV-infected women in this area.

Based on our results, approximately half of the HIV-infected women carried the HPV genome in their cervical mucosa. In a study in China involving 95 women infected with HIV, a DNA hybridization assay identified close to 43% women with at least one type of high-risk HPV infection (16). Another study in Brazil conducted on 634 HIV-infected women revealed the prevalence of HPV infection to be 48%, of which 94% were infected with a high-risk HPV (17), whereas another report from Brazil showed a higher prevalence of HPV (78.8%) among HIV-positive women (18). Previous reports suggest an increased prevalence of high-risk HPV types observed among HIV patients, compared to the HIV-negative patients (19). Given the high prevalence of HPV among the HIV patients in the present study, the existence of some cases with a co-infection with high-risk HPV genotypes is possible. As revealed in our study, HPV prevalence increased significantly by age, most probably because of a greater exposure to the infectious agents as the women grew older and had increased number of sexual partners. Moreover, none of the studied patients had history of HPV vaccination. Thus, it may be concluded that performing intensive HPV vaccination programs among younger populations in this region may help to decrease rate of infection in the future.

HPV infection, particularly of the high-risk HPV types, can be considered as a risk factor for developing CT and NG infections (20, 21). Our data showed a high prevalence of CT infection among the patients (38%); although none was suffering from Lymphogranulomavenereum (LGV) when they initially participated in the study. Earlier studies indicate that the time between the first intercourse and STI onset is very short (22). The high prevalence of CT in our patients is indicative of the presence of untreated asymptomatic persistent infections or repeated episodes of infections following inadequate treatment. In addition, no significant difference in CT frequency was observed between patients of different age groups. However, previous studies have demonstrated that the CT prevalence reduced with age, which could be due to repeated infections and immune response to the presence of the microorganism among older women. Franceschi et al. showed that CT prevalence was significantly higher among 15–24 year-old women compared with women between 24–30 years old (20). The discrepancy between present findings and other reports in terms of prevalence rates and correlations with age can be explained by the difference in the study population, i.e. the entire population in our study were HIV positive.

In the present study, 5.6% of the patients were diagnosed with NG. Similar studies among HIV-infected and non-infected women have reported different NG prevalence worldwide. The prevalence of NG among symptomatic women from different nationalities in Dubai, UAE was 5.5% (23). Previous studies in China revealed the prevalence rate to be between 1.8–37.8% among female sex workers (24). Another study among women living with HIV in Denmark showed that none were infected with NG (25). However, the study conducted in Papua New Guinea indicated a high prevalence of NG with the rate of 14.2% among the studied population (26). It has been observed that NG is becoming increasingly resistant to antibiotics worldwide, particularly among patients who receive repeated treatments for new infections (27, 28). Since the participants in the present study were among the high-risk groups with underlying diseases, i.e. HIV positive, it is very likely that they harbor emerging resistant strains and they may also serve as a reservoir for infecting others. Thus, development of comprehensive guidelines for screening and treatment of such STIs among the HIV-infected population seems to be vital.

In the present study, HSV DNA was detected in 10% of the HIV-infected women. In a similar study conducted on 369 HIV-positive women, more than 80% were observed to be HSV2 seropositive, and HSV DNA was detected in 7% of the respective cervico-vaginal samples (29). In a different study on 379 HIV-/HSV2- positive women, it was observed that 7% had viral secretion with a median load log 10 = 4.4 in their cervical os (30). HSV infection is common among HIV-positive women and is associated with an increased risk of HIV transmission. This observation might stem from the epithelial damage caused by HSV infection and consequently the increased shedding of HIV-infected cells and greater chance of HIV transmission to healthy individuals (31). Thus, with consideration of high prevalence of HSV infection (32), preventive strategies as well as appropriate HSV treatment (33) should be considered among such populations.

In the present study, 22.5% of the patients were infected with more than one agent. A previous study, conducted by the National Chlamydia Screening Program in London and its outskirts, showed that close to 3% of CT cases were infected with NG as well, but here men accounted for 1/3 of the study population (34). A Korean study on 709 women, referred to the hospitals for a general check-up for 6 common STIs, revealed that the co-infection rate was 6.8%; however, HPV and HSV were not addressed in this study (35). The relatively high rate of STI co-infection, similar to STI mono infection among the population in the present study, may be associated with their educational and socio-economic status. Educational status seems to play an important role in prevention, distribution and treatment of STIs (36, 37). Here, the majority of the studied patients had lower than high school education (80.2%) with a significant difference between STI-harboring and non-STI patients.

It has also been reported that the prevalence of STIs is higher in people having sexual contact with multiple sex partners, especially among high risk populations (38, 39). Our data also revealed that less than half of the studied patients had more than one sex partner (46.5%) and had been engaged in the practice of exchanging sex for money (29.6%). It was also shown that the prevalence of STIs was higher among illegal drug users; this may be due in part because they use sex as a way to financially support their drug habits (40). Although in the present study, all individuals with drug addiction (18.3%) belonged to the STI group, no statistical difference was found between STI-harboring and non-STI patients. Thus, the high prevalence of STIs among this population may be related to their legal male partners.

It has been shown that the STIs can be transmitted to adults during unprotected sex with infected sex partners, and the use of condoms can notably reduce the rate of such transmission (25, 39). We did not find any significant difference in condom use rate between STIs and non-STI patients, and the majority of the studied population declare that they almost always use condoms (87.3%). However, this known preventive strategy should be promoted steadily in the society, especially among the youth. As for the patients in the present study, the existence of any STIs in addition to risky sexual behaviors such as having multiple sex partners may result in an increase in the incidence of STIs in the region.

In conclusion, the present study identified a high prevalence of STIs among HIV-infected women in the region of Fars Province, Iran. Moreover, it provides useful information regarding STI-related risk factors in this population, which can help policymakers and STI experts for future research and planning of health guidelines and policies. Significant attention should focus on providing a comprehensive sex education to the potential patients, including recommending the use of condoms. In addition, a screening program for CT and NG should be developed, and the patients who are positive for such bacterial infections must be fully treated. Finally, high prevalence of HPV indicates the urgent need to develop a nationwide vaccination program aimed at females in the 10–12 years old age group.
